# Supplementary material for: Inhibition of HDAC and Signal Transduction Pathways Induces Tight Junctions and Promotes Differentiation in p63-Positive Salivary Duct Adenocarcinoma
Source: Cancers (Basel). 2022 May 24;14(11):2584. doi: 10.3390/cancers14112584 (PMC9179926; doi:10.3390/cancers14112584)
Supplement: Supplementary file 1 [file cancers-14-02584-s001.zip › cancers-1645067-supplementary.pdf]

cancers

MDPI

Supplementary Materials: Inhibition of HDAC and Signal Transduction Pathways Induces Tight Junctions and Promotes Differentiation in p63-Positive Salivary Duct Adenocarcinoma

Masaya Nakano, Kizuku Ohwada, Yuma Shindo, Takumi Konno, Takayuki Kohno, Shin Kikuchi, Mitsuhiro Tsujiwaki, Daichi Ishii, Soshi Nishida, Takuya Kakuki, Kazufumi Obata, Ryo Miyata, Makoto Kurose, Atsushi Kondoh, Kenichi Takano and Takashi Kojima

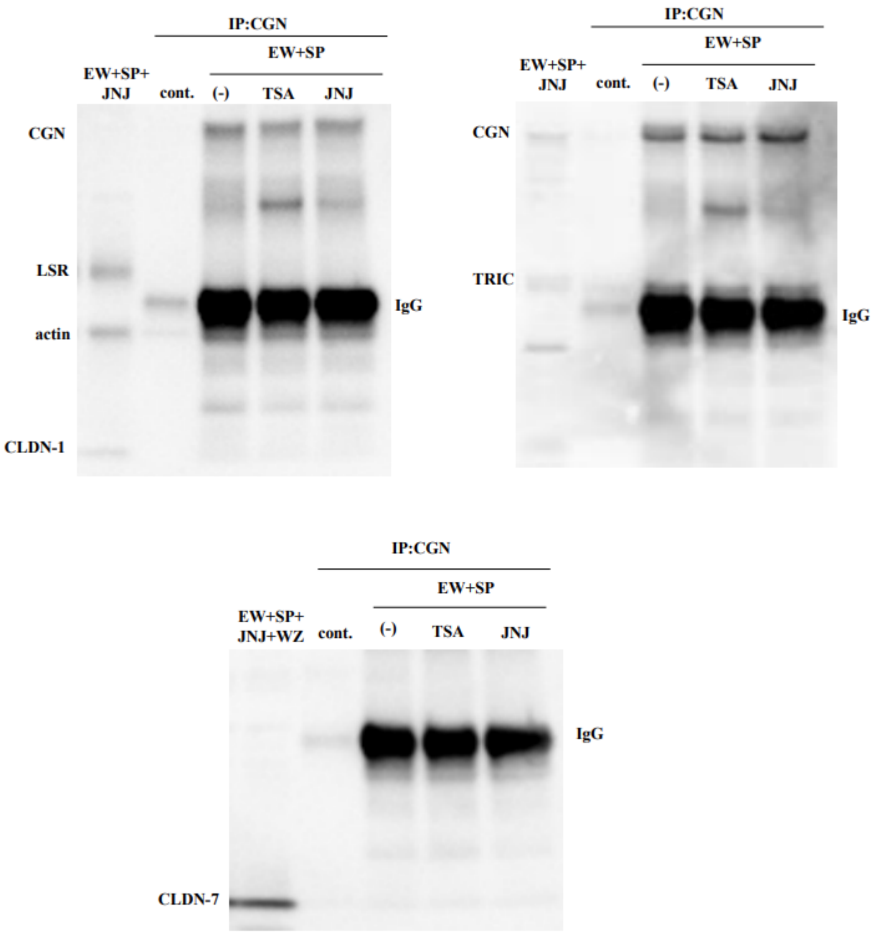

Figure S1. Coimmunoprecipitation in A253 cells treated with or without TSA and JNJ at 10 μM in the presence of EW-7197 and SP600125 at 10 μM. Immunoprecipitation using anti-CGN antibody led to the identification of angulin-1/LSR, TRIC, CLDN-1, CLDN-7 and actin in Western blot analysis.

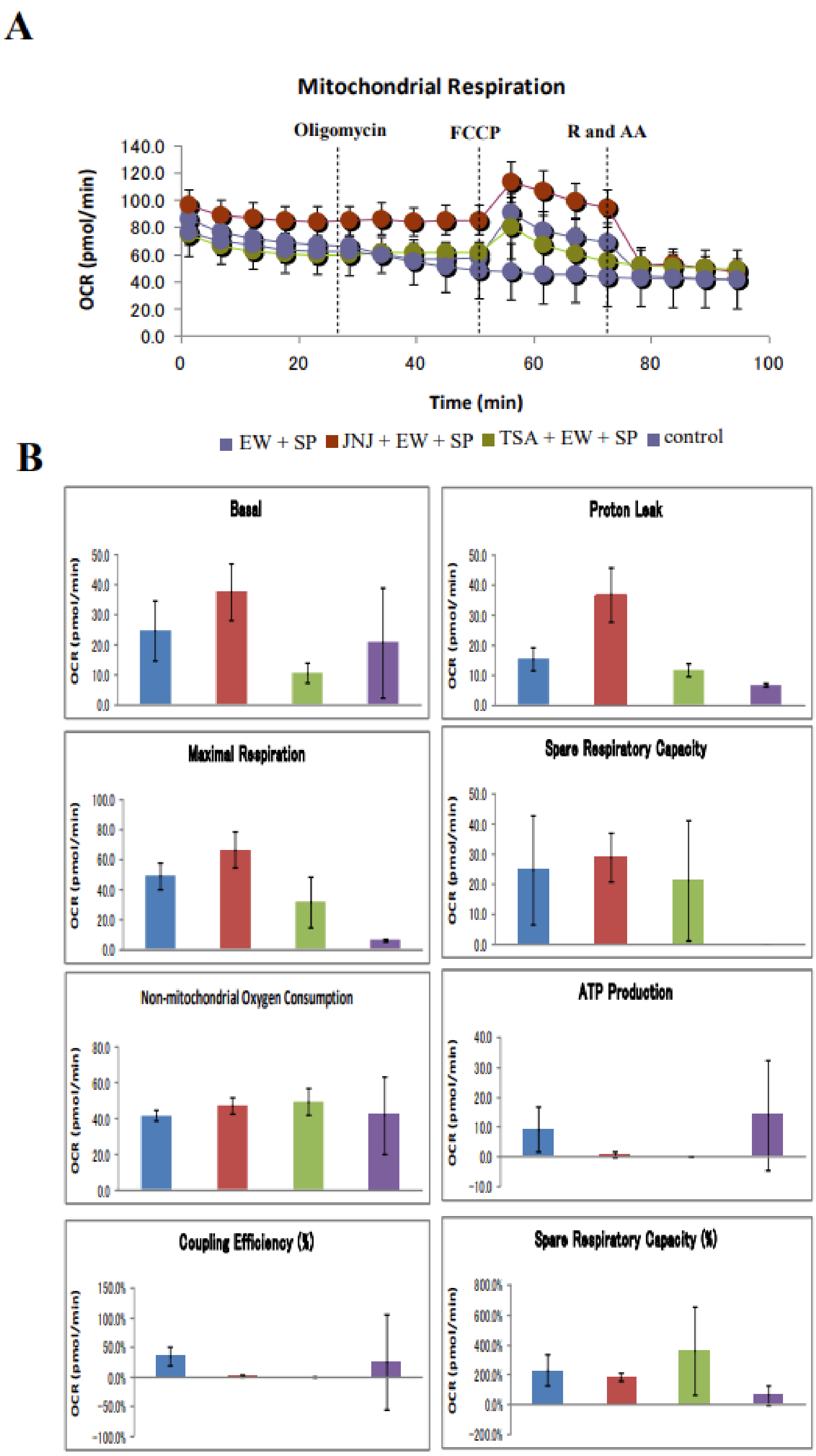

Figure S2. (a) Line graph and (b) bar graphs of OCR in A253 cells treated with or without TSA and JNJ at 10 µM in the presence of EW-7197 and SP600125 at 10 µM. Steady-state OCR was measured at six time points. Oligomycin was injected to inhibit ATP synthase, with the addition of FCCP to uncouple mitochondria and obtain the maximal oxygen consumption rate at the 8th time point. Finally, rotenone and antimycin A (R and AA) were injected to confirm that the respiration changes were due mainly to mitochondrial respiration.

Fig. 4A

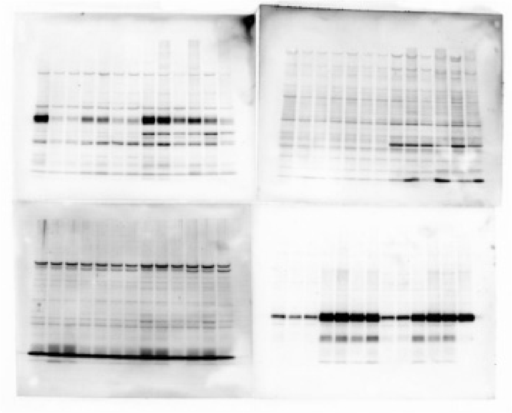

Fig. 5B

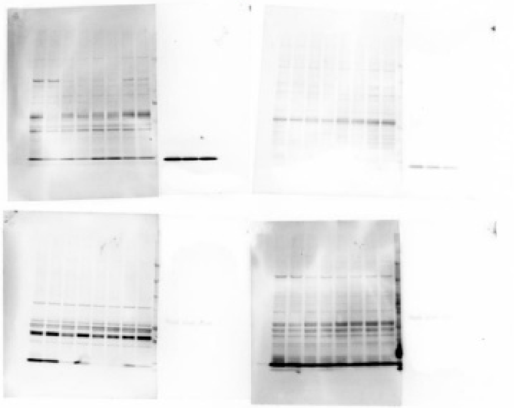

Fig. 4C and Supplemental Fig. 1

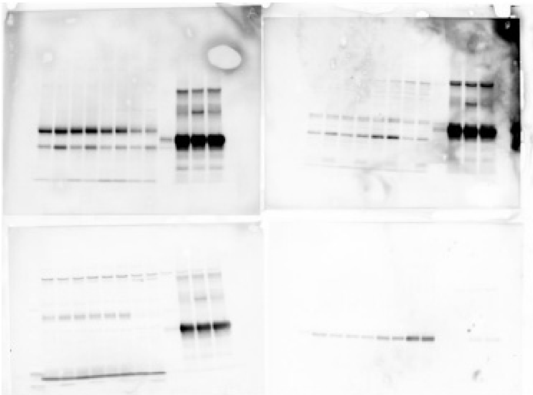

Figure S3. Original band images of all Western blotting.
